# Neuroprotective effects of niclosamide on disease progression via inflammatory pathways modulation in SOD1-G93A and FUS-associated amyotrophic lateral sclerosis models

**DOI:** 10.1016/j.neurot.2024.e00346

**Published:** 2024-03-15

**Authors:** Martina Milani, Ilaria Della Valle, Simona Rossi, Paola Fabbrizio, Cassandra Margotta, Giovanni Nardo, Mauro Cozzolino, Nadia D'Ambrosi, Savina Apolloni

**Affiliations:** a Department of Biology, University of Rome Tor Vergata, 00133 Rome, Italy; b Program in Cellular and Molecular Biology, University of Rome Tor Vergata, 00133 Rome, Italy; c Institute of Translational Pharmacology, CNR, 00133 Rome, Italy; d Department of Neuroscience, Istituto di Ricerche Farmacologiche Mario Negri IRCCS, 20156 Milan, Italy; e UniCamillus-Saint Camillus International University of Health Sciences, Rome, Italy

**Keywords:** Amyotrophic lateral sclerosis, FUS, Neuroinflammation, Niclosamide, SOD1, Transgenic mice

## Abstract

Amyotrophic lateral sclerosis (ALS) is a complex neurodegenerative disease influenced by genetic, epigenetic, and environmental factors, resulting in dysfunction in cellular and molecular pathways. The limited efficacy of current treatments highlights the need for combination therapies targeting multiple aspects of the disease. Niclosamide, an anthelminthic drug listed as an essential medicine, has been repurposed in clinical trials for different diseases due to its anti-inflammatory and anti-fibrotic properties. Niclosamide can inhibit various molecular pathways (e.g., STAT3, mTOR) that are dysregulated in ALS, suggesting its potential to disrupt these altered mechanisms associated with the pathology. We administered niclosamide intraperitoneally to two transgenic murine models, SOD1-G93A and FUS mice, mimicking key pathological processes of ALS. The treatment was initiated at the onset of symptoms, and we assessed disease progression by neurological scores, rotarod and wire tests, and monitored survival. Furthermore, we investigated cellular and molecular mechanisms affected by niclosamide in the spinal cord and muscle of ALS mice. In both models, the administration of niclosamide resulted in a slowdown of disease progression, an increase in survival rates, and an improvement in tissue pathology. This was characterised by reduced gliosis, motor neuron loss, muscle atrophy, and inflammatory pathways. Based on these results, our findings demonstrate that niclosamide can impact multiple pathways involved in ALS. This multi-targeted approach leads to a slowdown in the progression of the disease, positioning niclosamide as a promising candidate for repurposing in the treatment of ALS.

## List of abbreviations

ALS: Amyotrophic Lateral Sclerosis
fALS: Familial Amyotrophic Lateral Sclerosis
sALS: Sporadic Amyotrophic Lateral Sclerosis
SOD1: Superoxide Dismutase 1
TARDBP: Transactive Response DNA Binding Protein 43 ​kDa
FUS: Fused in Sarcoma
STAT3: Signal Transducer and Activator of Transcription 3
mTOR: Mammalian Target of Rapamycin
NF-kB: Nuclear Factor-kappa B
TDP43: TAR DNA-binding protein 43
WT: Wild Type
IP: Intraperitoneal
PFA: Paraformaldehyde
GCM: Gastrocnemius Muscle
CSA: Cross-Sectional Area
MyHC: Myosin Heavy Chain
IFNγ: Interferon-gamma
AUC: Area Under the Curve
AChRγ: Acetylcholine Receptor γ-subunit
GFAP: Glial Fibrillary Acidic Protein
Ntg mice: Non-transgenic mice

## Introduction

ALS is a complex neurodegenerative disease influenced by a combination of genetic, epigenetic, and environmental factors. This complex interplay of factors leads to dysfunction in various cellular and molecular pathways, contributing to the development and progression of the disease. The multifactorial nature of ALS may explain the limited success achieved by treatments proposed thus far, emphasising the necessity for multi-target therapies that can act synergistically on various aspects of the disease. About 10% of amyotrophic lateral sclerosis (ALS) cases are familial (fALS), with a family history, while the remaining 90% are sporadic (sALS). The first identified ALS-related gene was SOD1 approximately three decades ago [1]. However, in the last 15 years, several new genes associated with ALS have been discovered, including TARDBP (transactive response DNA binding protein 43 ​kDa), FUS (fused in sarcoma), and a hexanucleotide expansion repeat in Chromosome 9 Open Reading Frame 72 (C9ORF72) [2].

Niclosamide is an FDA-approved anti-helminthic drug used for over 50 years with considerable safety [3]. Niclosamide is a member of the salicylanilide class of pharmacologic agents with an aryl ß-hydroxy-carbonyl pharmacophore motif, usually present in many diverse biological natural products, that confers to this small molecule its pleiotropic biological activities and the potential to interact with multiple biological targets. Recently, niclosamide has been repurposed for diﬀerent diseases. Preclinical validation proved that niclosamide has eﬃcacy against solid cancers, rheumatoid arthritis, and ﬁbrotic conditions [4], and it is currently in phase II clinical trial for metastatic colorectal cancer [5] (NCT02519582), prostate cancer [6] (NCT02807805) and COVID-19 [7] (NCT04753619). The justification for utilizing niclosamide in treating neurological diseases has also been put forth in the literature [8]. This rationale is supported by the fact that niclosamide is a small lipophilic molecule, making it highly likely to penetrate the blood-brain barrier. Additionally, it is not a substrate for glycoprotein P, a significant hurdle in drug delivery to the central nervous system (CNS), further bolstering its potential as a therapeutic agent (www.drugbank.com).

It is well documented that niclosamide can inhibit diﬀerent molecular targets (STAT3, mTOR, Wnt/b-catenin, S100A4, SQSTM1/p62, NF-kB, Notch, TMEM16) [9,10] which are all dysregulated and pathogenic in ALS [[11], [12], [13]], suggesting its application to interfere with these altered mechanisms to improve multiple aspects of the pathology. Moreover, it has been demonstrated that niclosamide increases motoneuron diﬀerentiation and TDP43 clearance [14,15] and exerts neuroprotective eﬀects in peripheral neuropathies [16] and in models of Parkinson's disease [17,18].

We previously demonstrated that niclosamide inhibits microglia reactivity and migration; it reduces inﬂammation and ﬁbrosis and promotes autophagy in familial and sporadic ALS ﬁbroblasts [19]. Moreover, in a proof-of-concept experiment, niclosamide inhibited gliosis and ﬁbrosis in the spinal cord and in the sciatic nerve, promoted regeneration, and reduced ﬁbrosis and inﬂammation in the skeletal muscles of a small cohort of ALS mice [19].

Here, we have performed a preclinical validation of the drug by administering intraperitoneally niclosamide in two diﬀerent ALS mouse models, i.e., SOD1-G93A and FUS, that recapitulate ALS key pathological and biological processes [20].

## Methods

### Mice

Animal care procedures were conducted at the Tor Vergata University Animal Facility in accordance with the FELASA Recommendations, European Guidelines for the use of animals in research (2010/63/EU), and Italian Laws (D.L. 26/2014). The animals were housed in an indoor facility with a constant temperature of 22 ​± ​1 ​°C, relative humidity of 50%, and a 12-h light cycle (7 a.m.–7 p.m.). They had free access to food and water. Wet food was provided daily to the cages when the animals displayed signs of paralysis to ensure easy access to nutrition and hydration.

The SOD1-G93A mice used in the study were obtained from The Jackson Laboratory and were of the B6.CgTg (SOD1G93A)1Gur/J strain (#:004435). Transgenic hemizygous SOD1-G93A males were crossbred with C57BL/6 females, both maintained on a C57BL/6 genetic background. Genotyping of the transgenic progeny was performed using tissue extracts from tail tips. Briefly, the tail tips were digested overnight at 55 ​°C in tail buffer (100 ​mM Tris-HCl, pH 8, 0.5% Tween 20, 0.5% NP40) supplemented with 200 ​μg/mL Proteinase K. The samples were heated at 75 ​°C for 20 ​min to inactivate Proteinase K. The presence of the SOD1-G93A mutant transgene was determined by PCR using BioMix Red (Bioline) and the following primers: SOD1 forward 5′ CATCAGCCCTAATCCATCTGA 3′ and SOD1 reverse 5′ CGCGACTAACAATCAAAGTGA 3′.

The adult Tg (Prnp-FUS) WT3Cshw/J mice expressing hemagglutinin-tagged human wild-type FUS (FUS) were obtained from Jackson Laboratories. These mice were maintained as hemizygotes on a C57BL/6 genetic background. Hemizygous FUS mice were backcrossed to obtain homozygous mice, which were used as experimental subjects. Genotyping of the mice was performed through PCR analysis of tissue extracts from tail tips. Hemizygous FUS mice were identified using the following PCR primers: Fwr5′-AGGGCTATTCCCAGCAGAG-3′, Rev5′-TGCTGCTGTTGTACTGGTTCT-3′. Homozygous FUS mice were genotyped using qPCR with the following primers: Fwr5′-GCCAGAACACAGGCTATGGAA-3′ and Rev5′-GTAAGACGATTGGGAGCTCTG-5′.

All animal experiments were conducted in compliance with the ARRIVE guidelines and adhered to the European Guidelines for the use of animals in research (2010/63/EU) and the requirements of Italian laws (D.L. 26/2014). The ethical procedure was approved by the Italian Ministry of Health. Every effort was made to minimise animal suffering and reduce the number of animals used to obtain reliable results.

### Niclosamide *in vivo* treatments

A group of n ​= ​34 male SOD1-G93A mice, aged 13 weeks, were included in the study. They were treated intraperitoneally daily with either niclosamide at doses of 20 ​mg/kg or 50 ​mg/kg, doses previously established for mice [19,[21], [22], [23]], dissolved in 10% Cremophor® (vehicle) or with the vehicle. Treatment was initiated at the onset of the first symptoms and continued until the end phase of the disease (about 160 days of age) or until the mice reached 130 days of age (symptomatic phase) [24].

Additionally, a group of n ​= ​20 female SOD1-G93A mice, aged 13 weeks, were included in the study. They were treated intraperitoneally daily with niclosamide at a dose of 20 ​mg/kg dissolved in 10% Cremophor® (vehicle) or with the vehicle. Treatment was initiated at the onset of the first symptoms and continued until the end phase of the disease (about 160 days of age).

Similarly, a total of 32 sex matched FUS mice, aged four weeks, were included in the study. These mice were treated intraperitoneally daily with either niclosamide at a dose of 50 ​mg/kg dissolved in 10% Cremophor® (vehicle) or with the vehicle. Treatment was initiated at the onset of the first symptoms and continued until the end phase of the disease (about 40 days of age).

### Mice survival, neurological scores, and motor studies

The behavioral scores of the SOD1-G93A and FUS mice were assessed to monitor the progression of the disease. The assessments began at 70 and 25 days of age, respectively, for the SOD1-G93A and FUS mice. A rating scale ranging from 5 (representing a healthy mouse without symptoms of paralysis) to 1 (indicating full paralysis of the hind limbs, with the inability to straighten up within 30 ​s after being turned on their back) was used [25]. Once a mouse reached a score of 1, it was euthanised following preclinical testing guidelines [26].

To evaluate neuromuscular deficits, the mice underwent hanging wire tests twice a week, starting at 70 (SOD1-G93A) or 25 (FUS) days of age. The test involved placing the mouse on a wire grid (with a wire thickness of 2 ​mm) and gently shaking the grid to prompt the mouse to hold onto it. The grid was then turned upside down, and the latency for the mouse to release the grid was recorded within three attempts. The maximum recording time was 90 ​s for SOD1-G93A mice or 60 ​s for FUS mice [27,28]. Whenever a mouse dropped from the grid in three consecutive trials, it was considered symptomatic. For SOD1-G93A mice, motor performance was assessed twice a week using a rotarod apparatus (Ugo Basile 7650 model) set at a constant speed of 15 rotations per minute. The testing started at 11 weeks of age and continued until the mice could no longer remain on the rotarod. Following a three-day training period, the latency to fall off the rotarod was recorded as a measure of motor function. Each test day included three trials, and the best performance was recorded and included in the data analysis [29].

### Niclosamide pharmacokinetic studies

Niclosamide levels in plasma, brain, and spinal cord samples were quantified in 10-week-old C57BL/6 mice, following intraperitoneal (IP) administration at 20 ​mg/kg, as described in Table 1.

**Table 1:** Pharmacokinetic studies protocol.

Mouse IP protocol	
Species	Naïve mixed-gender C57Bl6 mice (n ​= ​9)
Route of administration (ROA)	IP
Animals per ROA	9
Dose level (mg/kg)	20
Dose volume (mg/ml)	2
Vehicle	10% Cremophor EL (BASF) and 0.9% NaCl
Sampling time	1 ​h (1/2/3), 6 ​h (4/5/6), 24 ​h (7/8/9)
Matrix for analysis	K_3_-EDTA plasma; brain; spinal cord
Data delivery	PK parameters: AUC_0-f_, AUC_0-inf_ and K_puu_

### Plasma, brain and spinal cord collection and treatment

Within a maximum of 15 ​min from the collection, blood was centrifuged at 4 ​°C using a Heraeus MultifugeR, set at 2200×*g* for 10 ​min. Then, plasma was transferred to appropriate tubes (Micronic®). The whole brain was explanted, washed with refrigerated saline solution (4 ​°C), divided into two halves, and weighed. The spinal cord was removed with the aid of a hook, washed with refrigerated saline solution (4 ​°C), and weighed. All samples were stored at −80 ​°C until analysis.

### Sample workup

Sample preparation of plasma, brain homogenate and spinal cord homogenate was based on protein precipitation with acetonitrile (matrix to organic solvent ratio 1 to 4 (v/v) or w/v), evaporation of the supernatant with N_2_ to dryness and reconstitution with H_2_O/acetonitrile 98/2 containing Warfarin and Labetalol as internal standards at a fixed concentration. Homogenisation of brain and spinal cord samples was performed by adding 1/3 (w/v) of artificial plasma (0.2 ​g of BSA in 50 ​mL of PBS 1×) and by using the Precellys® tissue homogenising (Bertin Technologies) at 5000 ​rpm, one cycle of 10 ​s. Calibration standard and quality controls for quantifying niclosamide in plasma, brain and spinal cord were prepared in artificial plasma. The samples were quantified using a calibration curve of 10 standards ranging from 0.0001 to 1 ​μM and four quality controls (ranging from 0.0005 to 1 ​μM), which were run in duplicate and analyzed against the calibration curve.

### Analytical method

The LC-MS/MS analytical method was developed using a Waters Acquity® UPLC system coupled with a Sciex API5000® triple quadrupole mass spectrometer using an ESI interface in MRM positive ion mode. An Acquity® UPLC HSS T3 1.8 ​μm, 2.1 ​× ​50 ​mm column was used at 40 ​°C, at a flow rate of 0.7 ​mL/min and an injection volume of 5 ​μL. The mobile phases were water 0.1% HCO_2_H (Phase A) and acetonitrile 0.1% HCO_2_H (Phase B), and the gradient profile was the following:

**Table:** 

Time (min)	% A	% B
Initial	95	5
0.50	95	5
2.00	50	50
2.10	2	98
2.40	2	98
2.41	95	5
3.20	95	5

Analyst® software 1.7.1 (AB SCIEX) was used to run samples, integrate peaks, and generate quantitation files to import into Watson® LIMS (Thermo Fisher) to perform standard regression, data analysis and pharmacokinetic calculations.

### Western blot

Spinal cords and gastrocnemius muscles were dissected from a subset of animals in each group (n ​= ​3/4 per group) [25]. The tissues were then lysed in a homogenisation buffer containing 50 ​mM Tris HCl pH 7.4, 250 ​mM NaCl, 1 ​mM EDTA, 5 ​mM MgCl2, 1% Triton X-100, 0.25% Na-deoxycholate, 0.1% SDS, and a protease inhibitor cocktail from Sigma-Aldrich.

To process the lysates further, two rounds of sonication cycles were performed, each lasting 10 ​s. After sonication, the samples were incubated on ice and centrifuged at 15,000×*g* for 20 ​min at 4 ​°C. The resulting supernatants were quantified using the Bradford protein assay (Bio-Rad) to determine protein concentration. The samples were then resuspended in Laemmli Buffer and prepared for SDS-PAGE (sodium dodecyl sulfate-polyacrylamide gel electrophoresis) using a 10% gel (Sigma-Aldrich). The proteins in the samples were separated by SDS-PAGE and transferred to nitrocellulose membranes. The membranes were incubated with 5% skimmed milk for 1 ​h to block non-specific binding sites. Primary antibodies were then applied to the membranes and incubated overnight at 4 ​°C. Immunoblots were performed with the following primary antibodies: rabbit anti-phospho-mTOR (1:500, Cell Signaling), rabbit anti-STAT3 (1:500, Cell Signaling), mouse anti-GAPDH (1:10000, Calbiochem), rabbit anti-S100B (1:500, Sigma), rabbit anti-Iba1 (1:500, Wako). After washing, the membranes were incubated with HRP-conjugated secondary antibodies (diluted at 1:2500, Jackson ImmunoResearch) at room temperature for 1 ​h. Secondaries antibodies for WB were anti-rabbit (1:2500) and anti-mouse (1:5000) IgG peroxidase-conjugated from Bio-Rad Laboratories (Hercules, CA, USA). Chemiluminescent detection was performed using an ECL solution (Roche). Densitometry-based quantification and analysis were performed using ImageJ software to quantify the protein bands. The relative density of each identified protein band was calculated. GAPDH (glyceraldehyde 3-phosphate dehydrogenase) was used as a control to ensure equal loading of proteins in each gel lane.

### Immunofluorescence and confocal analysis

SOD1-G93A and FUS mice, along with age-matched control mice, were euthanised with CO2 and subsequently decapitated. The spinal cords were promptly extracted and fixed in a 4% paraformaldehyde (PFA) solution for 12 ​h. Following fixation, the spinal cords were immersed in a 30% sucrose solution in PBS for 24 ​h at 4 ​°C. Subsequently, the cords were sliced into 30 ​μm thick sections using a freezing cryostat. For immunofluorescence analysis, lumbar spinal cord sections from each group (n ​= ​3/4 animals per group) were initially blocked for 1 ​h in a solution of 10% normal donkey serum (NDS) in PBS with 0.3% Triton X-100. They were then incubated for three days at 4 ​°C with primary antibodies, which were appropriately diluted in a solution containing 2% NDS in PBS with 0.3% Triton X-100. Immunofluorescences were performed with the following primary antibodies: rabbit anti-P2Y12 (1:500, Alomone), rabbit anti-HA-FUS (1:500, Cell Signaling), mouse anti-SMI32 (1:1000, Covance), rat anti-CD68 (1:500, AbdSerotec), rabbit anti-phospho-TDP43 (1:500, Proteintech), mouse anti-glial fibrillary acidic protein (GFAP) (1:500, Cell Signaling), rabbit anti-Iba1 (1:500 Wako). Subsequently, the sections were exposed to the corresponding secondary antibodies in the same solution for 3 ​h at room temperature. Secondary fluorescent antibodies were Alexa-Flour 488-Donkey anti-rabbit (1:200), Cy3-Donkey anti-mouse (1:200), Cy3-Donkey anti-rat (1:200), Alexa-Flour 488-Donkey anti-mouse (1:200) from Jackson ImmunoResearch Laboratories (West Grove, PA, USA). Following two PBS rinses, each lasting 10 ​min, nuclei were stained with 1 ​μg/ml of DAPI (Sigma-Aldrich) for 10 ​min. Immunofluorescence images were analyzed using a LEICA TCS SP5 confocal microscope equipped with three lasers: Argon/2, HeNe543, and HeNe633. Images were captured under constant exposure time, gain, and offset settings. Digital image brightness and contrast were adjusted using the LAS AF software (Leica). Background subtraction was performed after defining a region of interest, and the average pixel intensity was calculated. All image quantifications were done using ImageJ software (NIH, Bethesda, USA).

### Nissl staining

The total number of motor neurons in the L3–L5 lumbar spinal cord segments of each mouse was determined by examining sequential sections. Each section had a thickness of 30 ​μm, and every eighth section was analyzed. To identify the Nissl substance in neuronal cells, the sections (n ​= ​6/each mouse) were stained using a 1% cresyl violet. Following staining, the sections were gradually dehydrated using alcohol ranging from 50% to 100%, cleared with xylene, and then coverslipped using Eukitt (Sigma-Adrich, Saint Louis, MO, USA). All sections were photographed using a Zeiss Axioskop 2 microscope at a magnification of 20×. The right and left ventral horns of each section were examined to determine the number of large neurons, with cell bodies measuring ≥200 ​μm^2^ and distinct cytoplasm containing a nucleus and nucleolus. This counting process was performed using Neurolucida software (MBF Bioscience, Williston, VT, USA). The counts from the six sections were then averaged for each mouse.

### Microglia and astrocytes morphological analysis

For morphological analysis, spinal cord sections were stained with GFAP or Iba1, and images were acquired using a confocal microscope. For each condition, about 50 random cells per section from at least six sections for each animal were counted and analyzed using ImageJ (NIH, USA). For the measurement of area (A, in μm^2^), perimeter (P, in μm) and transformation index (TI), Iba1-and GFAP-positive cell images were converted into binary replicas using thresholding procedures implemented by ImageJ. The TI, which is a measure of differentiated cell morphology, was determined according to Fujita et al. (1996) [30] using the following formula: [perimeter of the cell (μm)] 2/4π [cell area (μm 2)].

### RNA-seq

Following the manufacturer's instructions, total RNAs were extracted from lumbar spinal cords using the Direct-zol™ RNA MiniPrep kit (ZYMO RESEARCH). Total RNA was quantified using the Qubit 4.0 fluorimetric assay (Thermo Fisher Scientific). Libraries were generated from 125 ​ng of total RNA through the NEGEDIA Digital mRNA-seq research grade sequencing service (Next Generation Diagnostic srl), encompassing library preparation, quality assessment, and sequencing on a NovaSeq 6000 sequencing system utilizing a single-end, 100-cycle strategy (Illumina Inc.).

The raw data underwent analysis *via* the proprietary NEGEDIA Digital mRNA-seq pipeline (v2.0) provided by Next Generation Diagnostic srl. This pipeline includes a cleaning step involving quality filtering and trimming with bbduk, alignment to the reference genome (mm10) using STAR 2.6.0a, and gene counting with HTseq-counts 0.9.1. Subsequently, the raw expression data were normalized and analyzed using the NEGEDIA degsanalysis pipeline (v1.2.0) [26]. The bidirectional hierarchical clustering heatmap was generated using FunRich software (version 3.1.3) [28]. Gene set enrichment analysis was performed using Enrichr analysis tool (Kuleshov, NAR 2016) and Rosalind HyperScale architecture (OnRamp BioInformatics, Inc.). Data visualisation was achieved by SRPLOT web server (https://www.bioinformatics.com.cn/en).

### Muscles analysis

Gastrocnemius (GCM) 12-μM coronal serial or 20-μm serial longitudinal cryosections were harvested onto poly-lysine-coated glass slides (VWR International). To assess the cross-sectional area (CSA) and the fiber type, transverse muscle sections from GCM were subjected to the following steps: initial fixation in acetone for 10 ​min, air drying, and subsequent washing. To retrieve antigens, the slides were immersed in citrate buffer (1×; Dako S2369) at 80 ​°C for 15 ​min, followed by an additional 15 ​min at room temperature, and then washed. After blocking with a 10% solution of normal goat serum (NGS) in PBS for 1 ​h, the muscle slides underwent incubation with Goat anti-mouse FAB fragment (1:100; Jackson Immuno 115-007-003) for 45 ​min, followed by a washing step. Muscle sections were immunostained with primary antibodies: mouse anti-MyHC type 1 (1:10; DSHB BA-D5), mouse anti-MyHC type 2a (1:17; DSHB SC-71), mouse anti-MyHC type 2b (1:9; DSHB BF-F3), rabbit anti-laminin (1:100; Sigma L9393), and corresponding secondary antibodies: Alexa-Fluor 564-goat anti-mouse MIgG2b (1:500; Invitrogen A21144), Alexa-Fluor 488-goat anti-mouse MIgG1 (1:500; Invitrogen A21121), Alexa-Flour 647-goat anti-mouse MIgM (1:500; Invitrogen A21046), and Alexa-Fluor 405-goat anti-rabbit (1:500; Abcam ab175649). Images were obtained using a sequential scanning mode on an A1 Nikon confocal microscope equipped with NIS-Elements software, capturing at a 20× magnification. Subsequently, muscle sections were analyzed using the “MuscleJ” plug-in in the Fiji software, following established procedures as previously documented [31].

To assess the endplate denervation analysis, five serial sections (average ​∼ ​70 neuromuscular junctions) per animal were analyzed as previously described [32]. Muscle sections were stained with mouse anti-synaptic vesicle protein (SV2; 1:100; DSHB), mouse anti-neurofilament 165 ​kDa (2H3; 1:50; DSHB), followed by Alexa Fluor 647-goat anti-mouse (1:500; Invitrogen). α-Bungarotoxin coupled to Alexa Fluor 488 (1:500; Invitrogen) was then added and left for 2 ​h at room temperature. Innervation analysis was performed directly. Images for the innervation analysis were obtained with an Olympus virtual slide system VS110 (Olympus, Center Valley, PA, USA) at 20×-magnification in Z-Stack. The percentage of neuromuscular innervation was quantified in OlyVIA (Olympus) on the basis of the overlay between neurofilament (SV2/2H3) staining and α-BTX labeled endplates. Endplates were quantified as occupied when there was any neurofilament staining overlying the endplate and as vacant when there was no overlay.

### RT-PCR

RNA was extracted from GCM using TRIzol (Invitrogen) and purified with PureLink RNA columns (Life Technologies). These RNA samples were treated with DNase I and underwent reverse transcription using the High-Capacity cDNA Reverse Transcription Kit (Life Technologies). Real-time PCR analysis was conducted using the TaqMan Gene expression assay (Applied Biosystems) following the manufacturer's recommended protocols. The reactions were performed on triplicate cDNA specimens, employing 1× Universal PCR Master Mix (Life Technologies) and a 1× mix containing specific receptor probes (Life Technologies).

Relative quantification was determined by calculating the ratio between the cycle number (Ct) at which the signal crossed a threshold set within the logarithmic phase of the target gene and that of the reference β-actin gene (4310881E; Life Technologies). The results were used for a 2-ΔCt statistical analysis. The following probes were employed: nicotinic cholinergic receptor, gamma subunit (AChRγ) (CHRNG; Mm00437419_m1; Life Technologies), interleukin 1β (Il-1β; Mm00434228_m1; Life Technologies), interferon-γ (IFNγ) (Mm01168134_m1; Life Technologies).

### Statistics

Data are reported as mean ​± ​standard error of the mean (SEM). Statistical differences were verified by a two-tailed student's *t*-test if the normality test was passed or by the Mann–Whitney rank sum test if the normality test failed. One-way analysis of variance (ANOVA) followed by Post hoc Tukey's was used for multiple comparisons. The onset of neuromuscular impairment and the survival length were statistically evaluated by a log-rank test to compare probabilities. The Kaplan–Meier was used to analyze mice survival. The software package GraphPad Prism 9.0 (GraphPad Software, San Diego, CA, USA) was used for all statistical analyses, with significant differences for p ​< ​0.05. Animals were randomly used for experiments. The sample sizes were chosen by power analysis and based on similar experiments reported in our previous papers and papers published by other groups [25,[33], [34], [35], [36]].

## Results

### Niclosamide crosses the blood-brain barrier

**Fig. 1:**
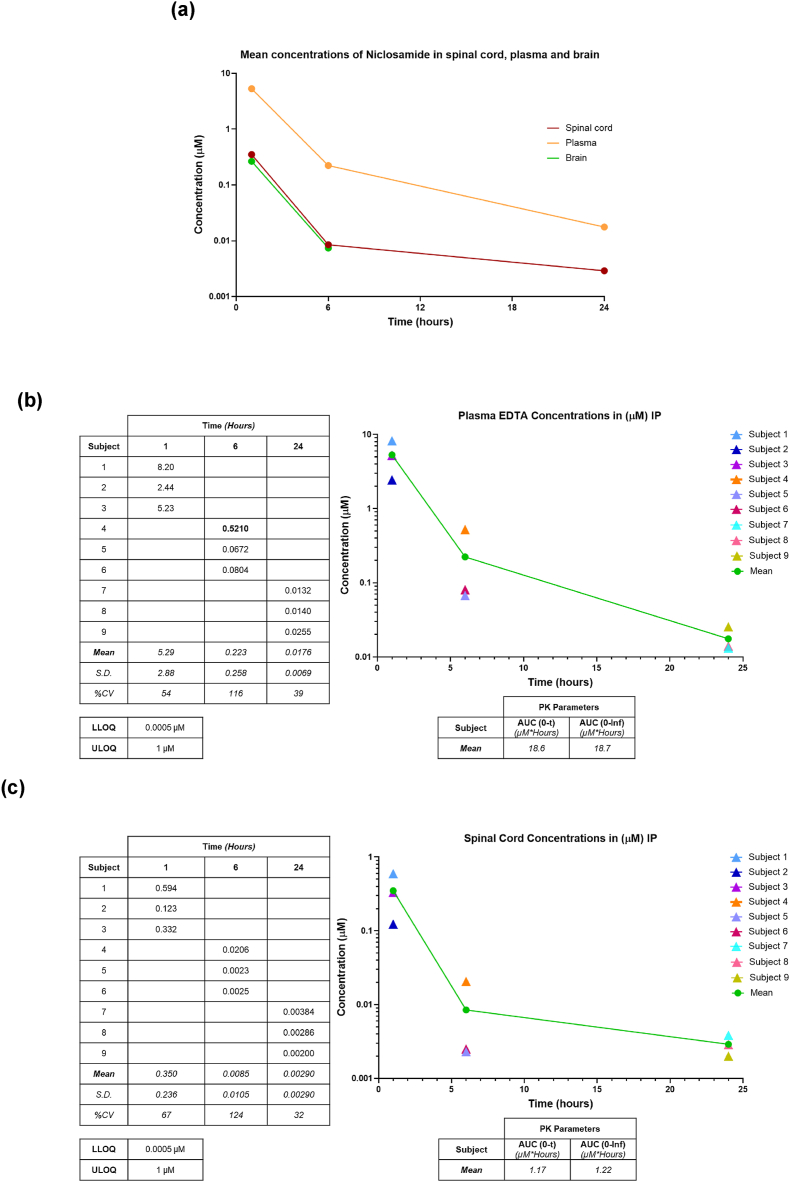
**Niclosamide crosses the blood-spinal cord barrier after intraperitoneal injections in mice**. (a) Niclosamide mean concentration levels (μM) in plasma, brain and in the spinal cord at 6-12-24 ​h after 20 ​mg/kg IP administration (n ​= ​3 mice/group). Pharmacokinetic details in plasma (b) and in spinal cord (c). AUC ​= ​area under the curve; LLOQ ​= ​lower limit of quantitation.

Due to the limited available data on the pharmacokinetics following intraperitoneal (IP) injection in mice, as well as the critical knowledge gap regarding the ability of niclosamide to penetrate the blood-brain barrier, our initial research focused on conducting comprehensive pharmacokinetic and brain penetration studies of niclosamide. Our findings reveal that niclosamide attains discernible concentrations in the plasma, brain, and spinal cord following IP injection, as illustrated in Fig. 1a. In plasma, the drug exhibits an average area under the curve (AUC) of approximately 18.6 ​mM, and even after 24 ​h post-injection, niclosamide remains detectable in the plasma (Fig. 1b). Our study demonstrates the capacity of niclosamide to traverse the blood-brain barrier (BBB), achieving noteworthy levels within the CNS with an average AUC of roughly 1.17 ​mM. Remarkably, niclosamide maintains its presence at nanomolar concentrations in the spinal cord 24 ​h post-injection (Fig. 1c).

### Niclosamide ameliorates disease progression and duration in SOD1-G93A mice

**Fig. 2:**
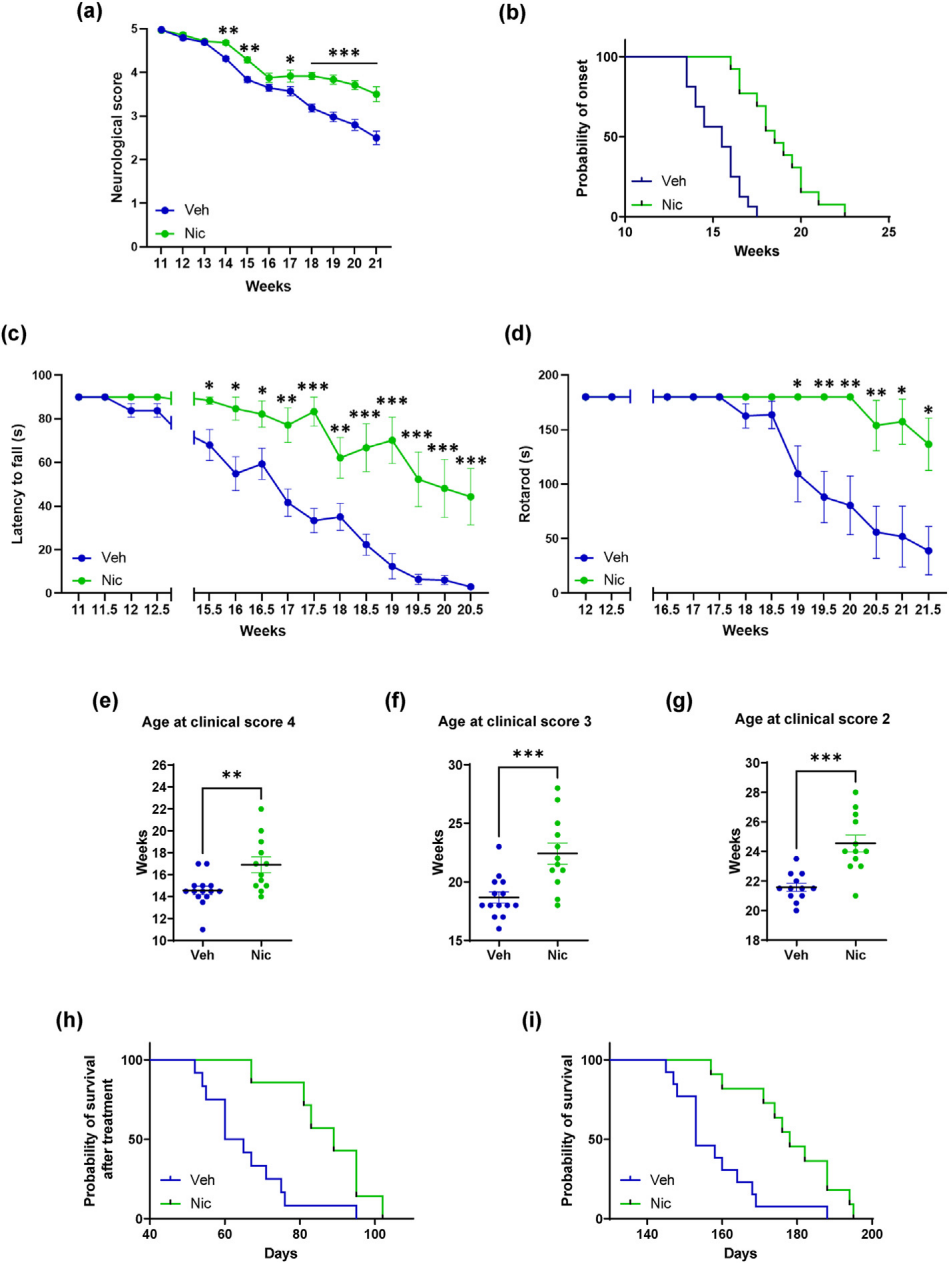
**Niclosamide ameliorates disease progression in SOD1-G93A mice**. Neurological scores (a), grip test (c) and rotarod performance (d) were significantly ameliorated in niclosamide 20 ​mg/kg (n ​= ​12, green) compared to vehicle-treated SOD1-G93A mice (n ​= ​14, blue). Data represent means ​± ​S.E.M. Statistical significance was calculated by ANOVA, ∗p ​< ​0.05, ∗∗p ​< ​0.01, and ∗∗∗p ​< ​0.001 Veh *vs* Nic; (b) Kaplan–Meier curve of SOD1-G93A mice showing increased time for the onset of neuromuscular deficits (time to reach 10% of neuromuscular impairment) evaluated by wire test following niclosamide treatments as compared to vehicle-treated mice. Statistical significance was calculated by log-rank test referred to vehicle, ∗∗∗p ​< ​0.001. The age at clinical score 4 (e), clinical score 3 (f) and clinical score 2 (g) were significantly delayed in niclosamide (green) treated SOD1-G93A mice compared to vehicle (blue). Data represent means ​± ​S.E.M. Statistical significance was calculated by ANOVA, ∗p ​< ​0.05, ∗∗p ​< ​0.01, ∗∗∗p ​< ​0.001. Kaplan–Meier survival curves of SOD1-G93A mice showing increased survival after the start of niclosamide treatment (h), and the overall survival (i) in niclosamide group as compared to vehicle group. Statistical significance was calculated by log-rank test referred to vehicle, ∗∗p ​< ​0.01.

We next analyzed how niclosamide affects pathology in the most common mouse model of ALS, SOD1-G93A mice, by administering niclosamide at the dose of 20 ​mg/kg dissolved in 10% Cremophor® (vehicle) or with the vehicle starting at the onset of first symptoms until end-phase. We demonstrated that niclosamide ameliorates male mice neurological scores (Fig. 2a). Niclosamide delays the onset of neuromuscular deficits (Fig. 2b), strongly ameliorates muscular strength, as shown by wire test, during all phases of the disease (Fig. 2c) and increases motor performances, as revealed by rotarod test (Fig. 2d). Niclosamide significantly increases the age at clinical score 4 (Fig. 2e), the age at clinical score 3 (Fig. 2f), and the age at clinical score 2 (Fig. 2g) of SOD1-G93A mice compared to vehicle-treated mice. Most importantly, niclosamide significantly improves by about 40% the probability of survival after treatment (62.5 *vs* 89 days, p ​< ​0.005 Fig. 2h) and by about 20% the overall survival of SOD1-G93A mice compared to vehicle-treated mice (155.5 *vs* 188 days, p ​< ​0.001 Fig. 2i).

In female SOD1-G93A mice, even though niclosamide at a dosage of 20 ​mg/kg only marginally improves disease progression (Fig. S1a-c), thereby indicating a gender-specific variation in its therapeutic effects, it does substantially enhance overall survival in SOD1-G93A mice compared to those treated with the vehicle alone (161 *vs* 174 days, p ​< ​0.01, Fig. S1d).

Finally, we administered niclosamide at 50 ​mg/kg to male SOD1-G93A mice. The results revealed that while this higher dose of niclosamide does lead to improvements in neurological scores (Fig. S2a), muscular strength (Fig. S2b), and motor performance (Fig. S2c), it does not result in a statistically significant increase in the probability of survival for the SOD1-G93A mice when compared to those treated with the vehicle alone (Fig. S2d).

### Niclosamide ameliorates tissue degeneration in the spinal cord of SOD1-G93A mice

**Fig. 3:**
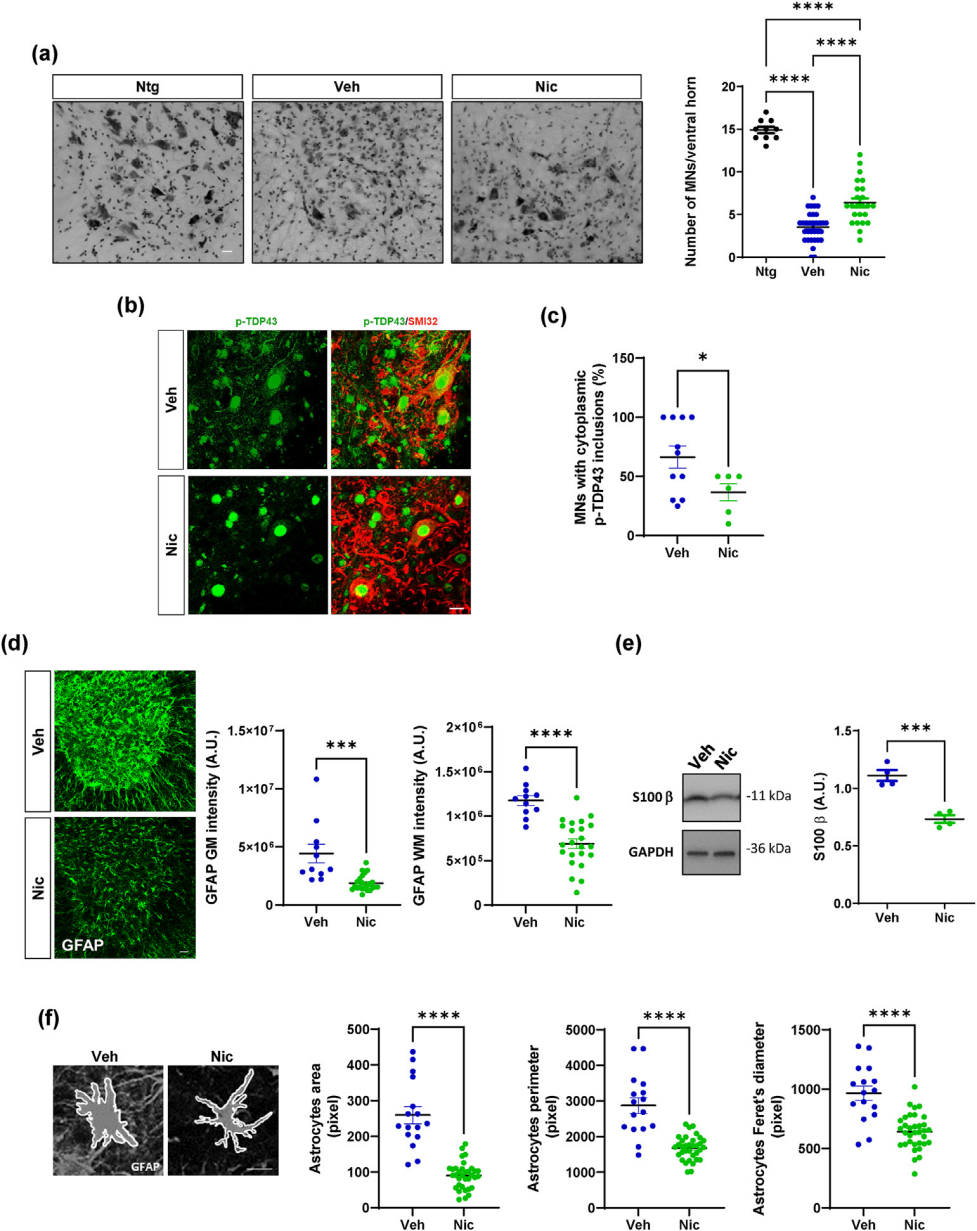
**Niclosamide decreases motoneuron loss, the cytoplasmic accumulation of p-TDP43 in the motoneurons and astrogliosis in lumbar spinal cord of SOD1-G93A mice**. (a) Nissl-stained spinal cord sections of Ntg (∼160 days) and terminal stage SOD1-G93A mice after vehicle or niclosamide 20 ​mg/kg treatment. Scale bar: 100 ​μm. Quantification of motor neuron (MNs) numbers/ventral horn is provided. Data represent means ​± ​S.E.M. Statistical significance was calculated by ANOVA ∗∗∗∗p ​< ​0.0001 (n ​= ​3/4 animals for group, at least four sections for animal). (b) Representative confocal images of motoneurons labeled with SMI32 (red) and p-TDP43 (green) in vehicle and niclosamide-treated SOD1-G93A mice. Scale bar: 10 ​μm. (c) Quantification of MNs with cytoplasmatic p-TDP43 inclusions in vehicle- and niclosamide-treated SOD1-G93A mice. (d) Representative confocal images of lumbar spinal cord sections of vehicle- and 20 ​mg/kg niclosamide-treated SOD1-G93A mice immunolabeled with GFAP (green). Scale bar: 100 ​μm. Quantification of GFAP staining in white matter (WM) and grey matter (GM) hemisections of both groups. Data are expressed as means ​± ​SEM. Statistical significance was calculated by *t*-test, ∗p ​< ​0.05. (n ​= ​3/4 animals for group, at least four sections for animal (e) Representative western blots and quantification of S100B in vehicle- and niclosamide-treated SOD1-G93A mice. GAPDH was used as a loading control. Data are expressed as means ​± ​SEM. Statistical significance was calculated by *t*-test, ∗p ​< ​0.05. (n ​= ​4 animals for group). (f) Astrocytes in vehicle- and niclosamide-treated SOD1-G93A mice were analyzed by ImageJ software for different size descriptors (Area, Perimeter and Feret's diameter). Scale bar: 50 ​μm. Data represent mean ​± ​S.E.M. (n ​= ​4 mice/group, at least 20 ​cells/mice). Statistical significance was calculated by *t*-test. ∗∗∗∗p ​< ​0.0001.

To analyze the cellular and molecular mechanisms targeted by niclosamide, SOD1-G93A male mice spinal cords were analyzed at the end stage of the disease. We demonstrated that niclosamide decreases motoneuron loss, as evidenced by the increase of motoneuron number/ventral horn in the treated group compared to the vehicle group (Fig. 3a). TAR DNA binding protein (TDP-43) mislocalization is a key pathological feature of ALS, and its redistribution to the cytoplasm of motor neurons in SOD1-G93A mice has been observed [37]. Remarkably, by analyzing the levels of the phosphorylated form of TDP43 protein in the cytosol of motoneurons, we demonstrated that niclosamide partially decreases the cytoplasmic accumulation of p-TDP43 protein in the motoneurons of treated mice compared to the vehicle group (Fig. 3b and c). Furthermore, we assessed the degree of gliosis and found compelling evidence that niclosamide significantly reduces astrocytosis in white and grey matter (Fig. 3d). Additionally, it leads to a decrease in the levels of the astrocytic marker S100B in the lumbar spinal cord of SOD1-G93A mice subjected to treatment (Fig. 3e). We next analyzed astrocyte morphological changes, revealing distinctive alterations in the niclosamide-treated group. Specifically, astrocytes displayed lower hypertrophy, smaller cell bodies, and thinner cellular processes than the vehicle-treated group (Fig. 3f).

**Fig. 4:**
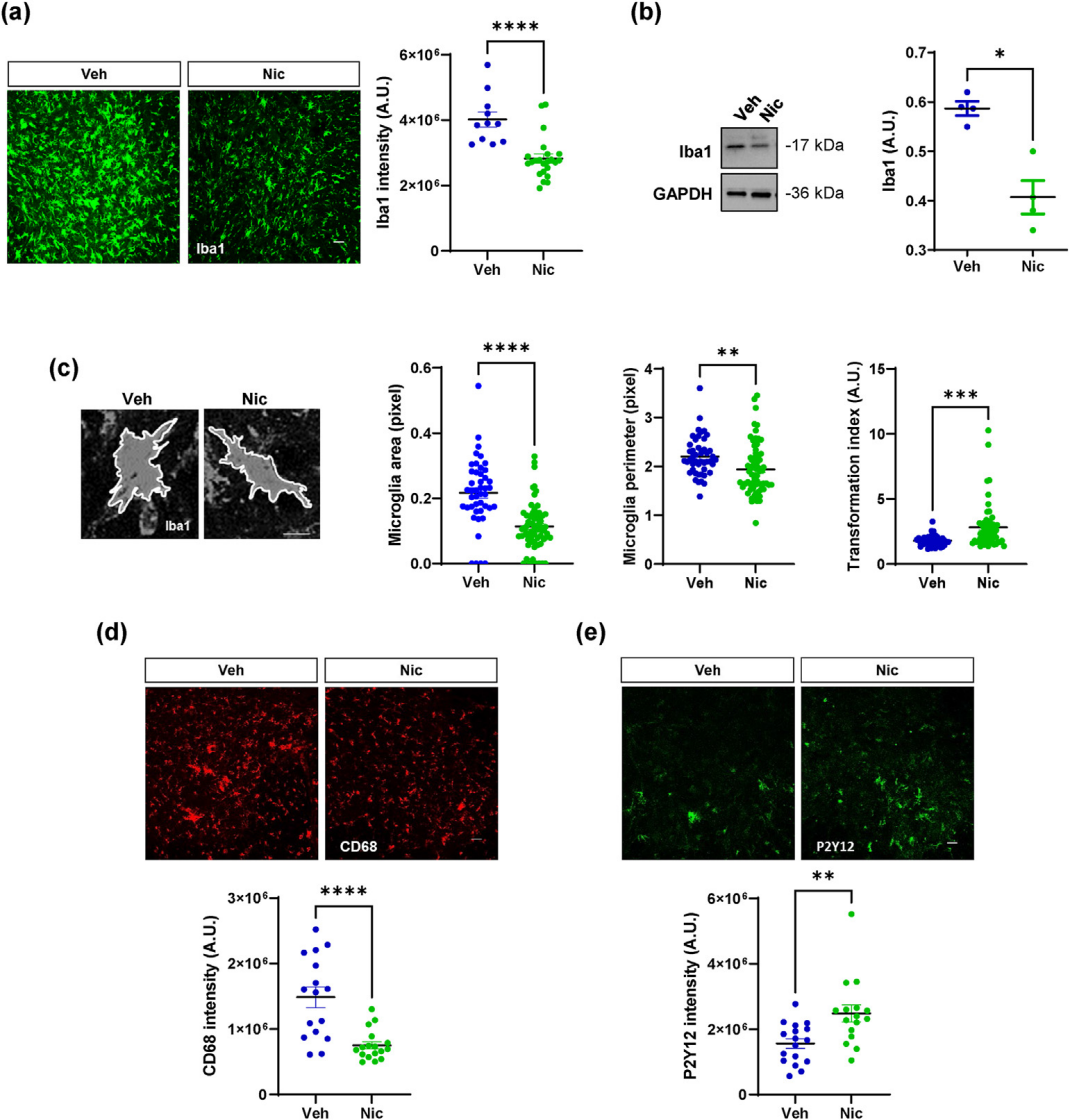
**Niclosamide decreases microgliosis and increase P2Y12-positive microglia**. (a) Representative confocal images of lumbar spinal cord sections of vehicle- and 20 ​mg/kg niclosamide-treated SOD1-G93A mice immunolabeled with Iba1 (green). Scale bar: 100 ​μm. Quantification of Iba1 staining in hemisections of both groups. Data are expressed as means ​± ​SEM (n ​= ​3/4 animals for group, at least four sections for animal). Statistical significance was calculated by student's *t*-test ∗∗∗∗p ​< ​0.0001. (b) Representative western blots and quantification of Iba1 in vehicle- (n ​= ​4) and niclosamide-treated (n ​= ​4) SOD1-G93A mice. GAPDH was used as a loading control. Data represent means ​± ​SEM. Statistical significance was calculated by student's t-test ∗p ​< ​0.05, (c) Microglia in vehicle- and niclosamide-treated SOD1-G93A mice were analyzed by ImageJ software for different size descriptors (Area, Perimeter). The transformation index was calculated as (perimeter)^2^/4πarea. Scale bar: 50 ​μm. Data represent mean ​± ​S.E.M. (n ​= ​4 mice/group, at least 20 ​cells/mice). Statistical significance was calculated by *t*-test. ∗∗p ​< ​0.01, ∗∗∗p ​< ​0.001. Representative confocal images of lumbar spinal cord sections cells labeled with anti-CD68 (d, in red) and anti-P2Y12 (e, in green) in vehicle- and 20 ​mg/kg niclosamide-treated SOD1-G93A mice. Scale bar: 100 ​μm. Quantification of CD68 (d) and P2Y12 (e) staining in hemisections of vehicle- and niclosamide-treated SOD1-G93A mice. Data represent means ​± ​S.E.M. Statistical significance was calculated by *t*-test, ∗∗p ​< ​0.01, ∗∗∗∗p ​< ​0.0001. (n ​= ​3/4 animals for group, at least four sections for animal).

In niclosamide-treated mice, we observed a robust decrease in microgliosis, as shown by the lower levels of Iba1 than in vehicle mice (Fig. 4a and b). Remarkably, microglia in niclosamide mice have smaller cell sizes and longer projections than those in vehicle mice, appearing more ramified, as evidenced by an increase in the transformation index value (Fig. 4c).

To deeply investigate the effect of niclosamide on microgliosis and infiltrating immune cells, we evaluated the levels of CD68 and P2Y12 protein, showing that niclosamide strongly decreases the expression of CD68 reactive microglia/macrophages (Fig. 4d) while increasing the expression of P2Y12 homeostatic microglia (Fig. 4e) in the spinal cord of SOD1-G93A treated mice.

Finally, niclosamide strongly inhibits the protein expression of its specific targets, STAT3 and mTOR, in the lumbar spinal cord of SOD1-G93A mice compared to the vehicle group (Fig. S3a, b).

### Niclosamide alters the transcriptomic profile of SOD1-G93A mice

**Fig. 5:**
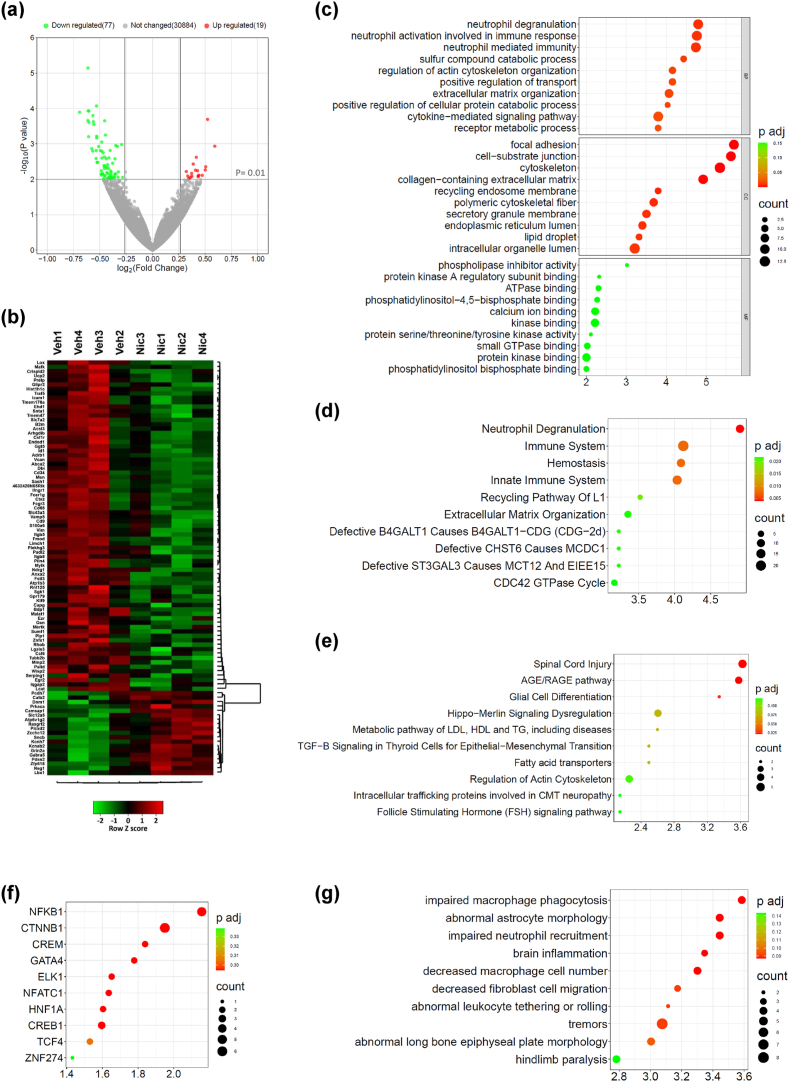
**Niclosamide alters the transcriptomic profile of SOD1-G93A mice spinal cord**. (a) Volcano plot of genes showing the magnitude (log2 (fold change), x-axis) and significance (−log10 (P value), y-axis) for niclosamide compared to vehicle SOD1-G93A mice (n ​= ​4 animals/group). The horizontal and vertical grey lines highlight statistical significance (p value ​< ​0.01) and fold change (FC) (FC ​> ​1.2) thresholds, respectively. Differentially expressed (DE) genes are marked in red (upregulated genes) or green (downregulated genes). (b) Hierarchical clustering heatmap of DE genes of niclosamide (nic) compared to vehicle (veh) SOD1-G93A mice. (c–g) Gene set enrichment analysis of DE genes between nic and veh mice. Top 10 enriched terms for Gene Ontology (c), Reactome (d), WikiPathway (e), Transcription Factor (f), Mammalian Phenotype Ontology (g) are displayed based on decreasing -log10 (p value) (enrichment). The colour code shows the adjusted p value, while the size of bubble represents the number of genes enriching the corresponding annotation (count). (c) BP, biological process; CC, cellular component; MF, molecular function.

To depict the overall transcriptome alterations occurring during disease progression and affected by niclosamide treatment, we analyzed the RNAs from the spinal cord of end stage niclosamide and vehicle-treated SOD1-G93A mice by RNA-sequencing. We found 94 differentially expressed genes (DEGs) in the niclosamide group, with a p-value <0.01 and fold change greater than 1.2. Among them, 75 display decreased expression, and 19 are upregulated with respect to the vehicle group (Fig. 5a and b, Table S1). Using Gene Ontology and pathway enrichment analysis, we observed that differentially expressed genes (e.g., *Icam1, Vim, Mmp2, CD68*) were related to categories such as “spinal cord injury”, “brain inflammation”, “decreased macrophages cell number”, “cell migration”, “aberrant astrocytes morphology” (Fig. 5c–g), all processes, functions and molecules related to the mechanism of action of the drug that could explain the observed therapeutic efficacy of niclosamide on disease progression.

Finally, we chose to analyze transcriptomic changes at the symptomatic phase of the disease, approximately 130 days of age. This phase was selected because it represents the average onset of motor neuron degeneration [38], and during this period, the differences between the niclosamide and vehicle groups exhibit significant significance in motor tests. Interestingly, by analyzing the RNAs from the spinal cord of niclosamide and vehicle-treated SOD1-G93A mice, RNA sequencing reveals that even at this disease stage, seven genes are significantly altered by niclosamide (Fig. S4a, Table S1), including *sgk1*, a gene involved in glia-mediated neuroinflammation [39].

### Niclosamide ameliorates skeletal muscle pathology in SOD1-G93A mice

**Fig. 6:**
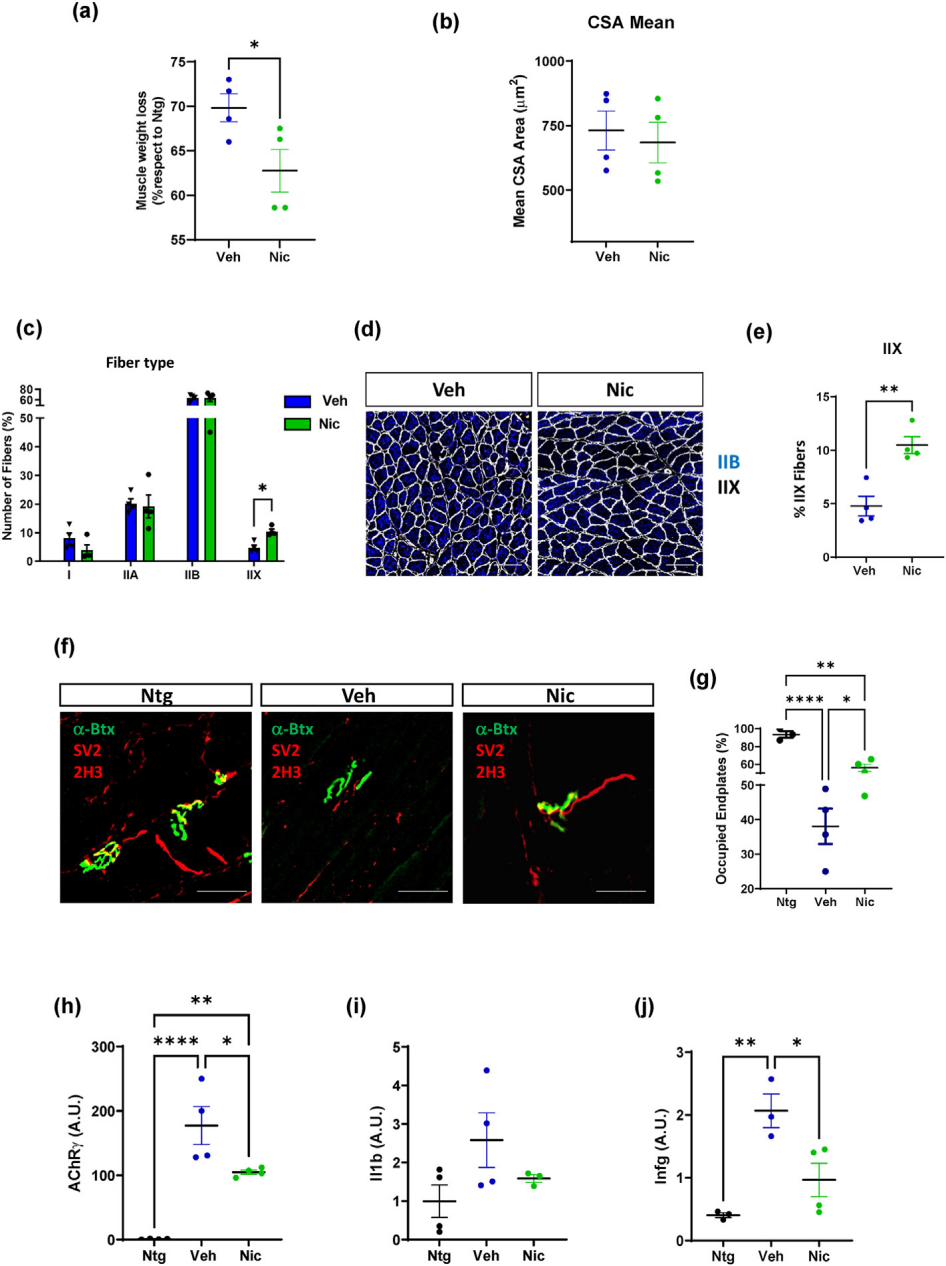
**Niclosamide promotes the generation of fast-fatigable IIX muscle fibers, delays muscle atrophy and sustains an anti-inflammatory state in the muscles of SOD1-G93A mice**. Muscle wasting was calculated by measuring the Gastrocnemius Medialis (GCM) (a) muscle weight of niclosamide and vehicle-treated SOD1-G93A mice compared to Ntg littermates. Mean cross-sectional area (CSA) of muscle fibers (b) and quantification of fiber-type content (c and d) in GCM muscle analyzed with the “MuscleJ” plug-in of Fiji software. (d-e) Representative immunofluorescent staining and quantification of muscle fiber typing via myosin heavy chain (MHC) isoforms; Corresponding color legend for fiber types: type IIX ​= ​unstained (black), type IIB ​= ​blue, and laminin ​= ​white. Staining was performed on GCM muscle sections obtained from vehicle and niclosamide treated mice. Analysis of denervation on GCM muscle of vehicle- and niclosamide-treated SOD1G93A mice is calculated by the percentage of occupied endplates (f and g). Real-time qPCR for AChRy (h), IL-1β (i) and IFNg (j) mRNA transcripts in GCM muscle of niclosamide and vehicle-treated SOD1-G93A mice compared to Ntg littermates. Data represent mean ​± ​SEM. Statistical significance was calculated by one-way ANOVA with Tukey's post-analysis, ∗p ​< ​0.05, ∗∗p ​< ​0.01, ∗∗∗p ​< ​0.001.∗∗∗∗p ​< ​0.0001 (n ​= ​3/4 animals for group).

Building upon the promising data regarding neuromuscular strength in the wire test, we analyzed the effect of niclosamide administration on metabolism and inflammatory status in the skeletal muscle of SOD1-G93A mice at the end stage of the disease. At first, the gastrocnemius muscle (GCM) was weighted to evaluate the niclosamide effect on muscle mass. Fig. 6a shows that muscles from niclosamide-treated mice exhibited reduced wasting compared to vehicle-treated mice. Albeit no difference was found in muscle fiber cross-sectional area (CSA) between vehicles and niclosamide-treated SOD1-G93A mice (Fig. 6b and c), the latter showed a higher percentage of fast fatigable glycolytic type IIX fiber subtype (Fig. 6d and e), suggesting a niclosamide-mediated reduced oxidative shift of muscle fiber, a common hallmark in ALS [40]. Consistently, the denervation of GCM muscles was lower in SOD1-G93A mice treated with niclosamide compared to the vehicle-treated group, as evinced by significant preservation of innervated endplates (Fig. 6f–h).

Following muscle denervation, transcription of nAChR subunits markedly increases, leading to the generation of brand new AChRs in which the epsilon (ε) subunit is mainly replaced by the fetal gamma (γ) subunit [41]. A significant decrease in fetal acetylcholine receptor γ-subunit (AChRγ) mRNA levels was observed after niclosamide treatment, indicating preservation of hindlimb muscle innervation compared to vehicle-treated mice (Fig. 6h). We next investigated the inflammatory environment within the skeletal muscle of SOD1-G93A mice following niclosamide administration. The outcomes showed a trend toward the downregulation of interleukin-1 beta (IL-1β) (Fig. 6i) and a significant reduction of interferon-gamma (IFN-γ) (Fig. 6j) in the GCM of niclosamide-treated mice versus the vehicles.

### Niclosamide ameliorates disease progression in FUS mice

**Fig. 7:**
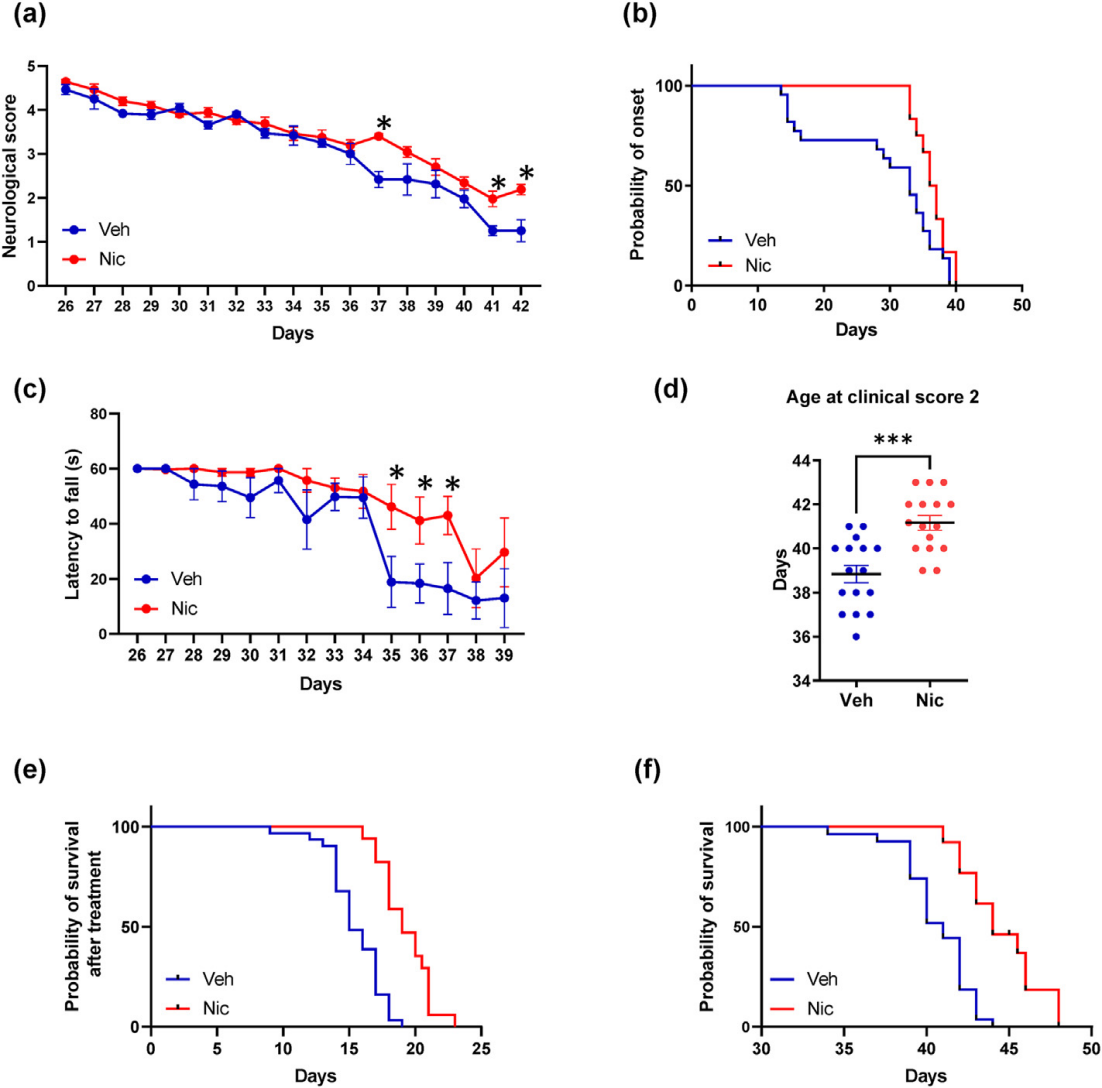
**Niclosamide ameliorates disease progression in FUS mice**. Neurological scores (a) and grip test (c) were significantly ameliorated in niclosamide 50 ​mg/kg (n ​= ​16, red) compared to vehicle treated FUS mice (n ​= ​16, blue). Data represent means ​± ​S.E.M. Statistical significance was calculated by *t*-test referred to vehicle, ∗p ​< ​0.05. (b) Kaplan–Meier curve of FUS mice showing increased time for the onset of neuromuscular deficits (time to reach 10% of neuromuscular impairment) evaluated by grip test following niclosamide treatment as compared to vehicle-treated mice. Statistical significance was calculated by log-rank test referred to vehicle, ∗∗p ​< ​0.01. (d) The age at clinical score 2 was significantly delayed in the niclosamide-treated FUS mice compared to vehicle. Data represent means ​± ​S.E.M. Statistical significance was calculated by *t*-test referred to vehicle, ∗∗∗p ​< ​0.001. Kaplan–Meier survival curves of FUS mice showing increased survival after the start of niclosamide treatment (p ​< ​0.01, e), and the overall survival (p ​< ​0.01, f) in niclosamide group as compared to vehicle group. Statistical significance was calculated by log-rank test referred to vehicle.

Based on the promising results obtained with niclosamide 20 ​mg/kg in ameliorating pathological hallmarks of the disease in FUS mice [19], in this work, we assessed disease progression and duration upon 50 ​mg/kg niclosamide treatment in FUS mice, demonstrating that the drug partially ameliorates neurological scores (Fig. 7a), delays the onset of neuromuscular deficits (Fig. 7b) and improves muscular strength as evinced by wire test (Fig. 7c). Remarkably, niclosamide significantly increases the age at clinical score 2 (Fig. 7d), the disease duration (15 *vs* 19 days, p ​< ​0.0001 Fig. 7e), and the overall survival of FUS mice with respect to vehicle-treated mice (41 *vs* 44 days, p ​< ​0.0001 Fig. 7f). No differences between male and female mice were recorded.

### Niclosamide ameliorates spinal cord pathology in hFUS mice but has no effect on muscle denervation atrophy

**Fig. 8:**
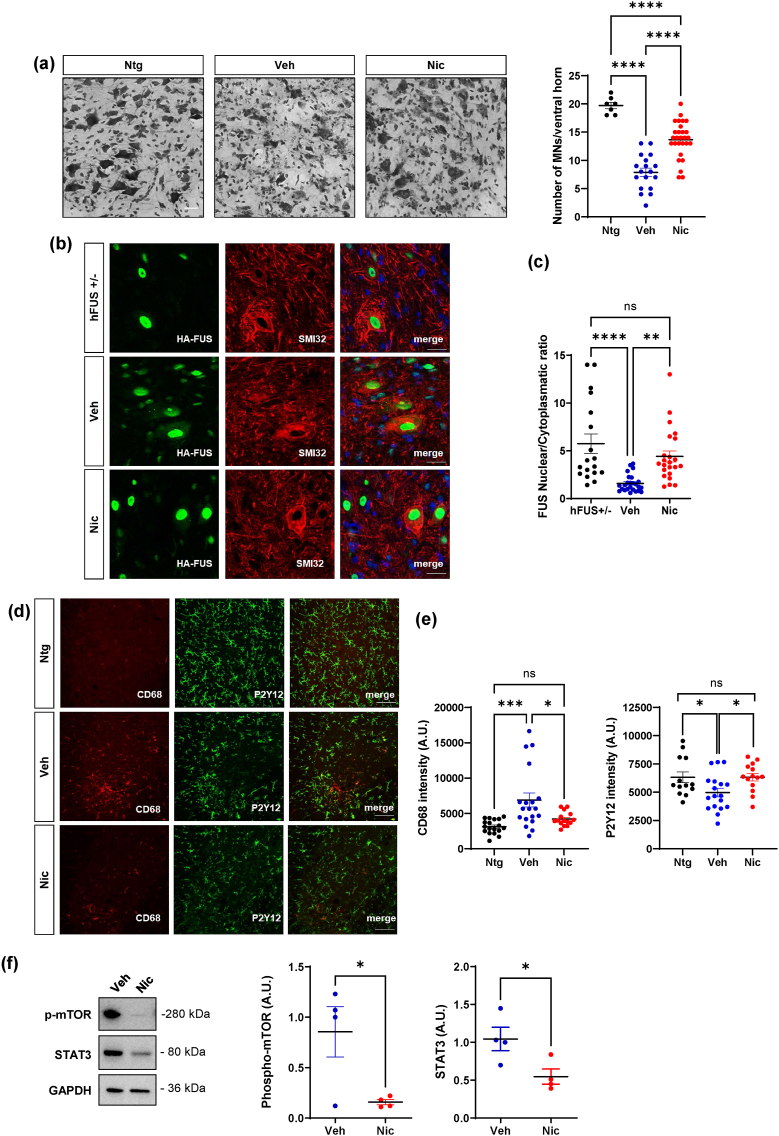
**Niclosamide ameliorates spinal cord pathology in FUS mice**. (a) Nissl-stained spinal cord sections of Ntg (∼40 days) and terminal stage FUS mice after vehicle or niclosamide 50 ​mg/kg treatment. Scale bar: 100 ​μm. Quantification of motor neuron (MNs) numbers/ventral horn is provided. (b) Representative confocal images of motoneurons labeled with SMI32 (red) and HA-FUS (green) in heterozygous FUS (FUS+/−), vehicle and niclosamide-treated FUS mice. Scale bar: 10 ​μm. (c) Quantification of FUS nuclear/cytoplasmatic ratio in FUS+/−, vehicle- and niclosamide-treated FUS mice. (d) Representative confocal images of lumbar spinal cord sections cells labeled with anti-CD68 (red) and anti-P2Y12 (green) in Ntg, vehicle- and niclosamide-treated FUS mice. Scale bar: 100 ​μm. (e) Quantification of CD68 and P2Y12 staining in hemisections of Ntg, vehicle- and niclosamide-treated FUS mice. Data represent means ​± ​S.E.M. Data represent means ​± ​S.E.M. Statistical significance was calculated by ANOVA, ∗p ​< ​0.05, ∗∗p ​< ​0.01, ∗∗∗p ​< ​0.001. (n ​= ​3/4 animals for group, at least four sections for animal). (f) Representative western blots and quantification of p-mTOR and STAT3 in vehicle- (n ​= ​4) and niclosamide-treated (n ​= ​4) FUS mice. GAPDH was used as a loading control. Data represent means ​± ​SEM. Statistical significance was calculated by student's *t*-test or by Mann-Whitney test referred to vehicle-FUS group, ∗*p* ​< ​0.05.

To assess the cellular and molecular mechanisms aﬀected by niclosamide in FUS mice, spinal cord was analyzed at the end stage of the disease. We demonstrated that niclosamide decreases motoneuron loss (Fig. 8a) in the spinal cord of treated mice compared to vehicle mice. Remarkably, by analyzing the levels of FUS in both nuclei and cytosol of motoneurons, we demonstrated that, while FUS mice showed an evident delocalization of the protein into the cytoplasm compared to healthy mice, niclosamide partially prevents the cytoplasmic mislocalization of FUS in the motoneurons, compared to the vehicle group (Fig. 8b and c). Moreover, we evaluated the extent of gliosis, demonstrating that niclosamide decreases microgliosis (Fig. S5a) but does not change the overall staining of GFAP-positive astrocytes (Fig. S5b) in the lumbar spinal cord of treated mice. To investigate thoroughly the impact of niclosamide on microgliosis and infiltrating immune cells, we analyzed the levels of CD68 and P2Y12 proteins, showing that, while FUS mice display higher CD68 staining and reduced P2Y12 signal than Ntg mice in the spinal cord, niclosamide decreases the expression of CD68 activated microglia/macrophages, and increases the expression of P2Y12 surveillant microglia (Fig. 8d and e). Finally, in keeping with data on SOD1-G93A mice, we demonstrated that niclosamide strongly inhibits the protein expression of its specific inﬂammatory/autophagic targets STAT3 and mTOR in the lumbar spinal cord of FUS mice compared to the vehicle group (Fig. 8f). Despite the positive effect observed in the spinal cord, the analysis of muscle mass and denervation showed no significant differences between the vehicle and niclosamide-treated groups (Fig. S6a and b).

## Discussion

While several drugs have shown promise in extensive clinical trials, few drugs have received FDA approval and are presently accessible as patient treatment choices [42]. The current research study provides evidence that administering niclosamide effectively mitigates a broad spectrum of disease features associated with ALS in both SOD1-G93A and FUS transgenic mouse models, characterised by different molecular disease determinants.

Previous studies in experimental models of neurodegenerative diseases have suggested that niclosamide may possess neuroprotective properties through various mechanisms [43], including promoting autophagy [17,44]. We showed that niclosamide reduces the cytoplasmic accumulation of phosphorylated TDP43 in the motoneurons of SOD1-G93A mice, a critical pathological feature observed in sporadic and familial forms of ALS [45]. Indeed, although the presence of total TDP43 aggregates was not initially reported in SOD1-related fALS nor in SOD1 mice [46], several recent studies have documented the presence of TDP43 inclusions in motoneurons from SOD1 patients, albeit to a lesser extent compared to other ALS cases [37,47,48]. Additionally, the phosphorylated form of TDP43 has been detected in SOD1 mice at both symptomatic and end stages of the disease [37]. The effect observed is in line with recent research demonstrating that niclosamide can rescue TDP-43 mislocalization induced by stressors in cultured iPSC-motor neurons [15] and hints at the investigation of the potential efficacy of niclosamide in the sporadic form of the disease, characterized by TDP43 pathology.

It is well known that the progressive symptoms of ALS result from the loss of motor neurons and dysfunctions in non-neuronal cells [49]. Our findings demonstrate that niclosamide can target critical cell phenotypes involved in ALS, including glial cells and skeletal muscles. Consistently, in the lumbar spinal cord of SOD1-G93A mice, niclosamide affects astrocytes and microglial morphology, which could be explained by a direct modulation of genes involved in cell cytoskeleton remodelling [36], and to the re-acquisition of a surveillant phenotype, as suggested by the increased expression of P2Y12 positive cells [50]. Interestingly, niclosamide reduces muscle wasting, likely contrasting the peripheral immune component of the disease [51] and acting as an anti-inflammatory drug both at CNS and muscle levels.

Nevertheless, in female SOD1-G93A mice, niclosamide had a more modest impact, resulting in only a slight increase in overall survival. Gender-related variations in treatment response are not uncommon in preclinical studies involving the SOD1-G93A model [52]. While the exact mechanisms behind these differences are not fully understood, they may be influenced by hormonal factors that affect disease progression [51,53]. It is important to note that differences in niclosamide effects linked to sex hormones have been observed between genders in other contexts [54]. Moreover, it is well known that females display different pharmacokinetics compared to males, with lower renal excretion and differences in the activity of liver enzymes, possibly explaining a higher incidence of adverse events observed in this gender for many FDA-approved drugs [55].

Notably, the preservation of muscle mass without a significant change in the CSA of muscle fibres could indicate specific adaptations of the muscle in response to the treatment. For instance, functional compensation mechanisms (e.g., neuromuscular efficiency; contractile force), changes in tissue composition (e.g., adipose and/or connective tissue) or metabolism might contribute to the preservation of muscle mass without a noticeable increase in CSA.

In addition, our study shows that in SOD1-G93A mice, 50 ​mg/kg niclosamide does not yield the same level of effectiveness as the lower dose (20 ​mg/kg) in terms of overall survival, even though it leads to improvements in rotarod performance and behavioral scores. It is important to note that niclosamide is generally well-tolerated in both humans and rodents and that it has shown minimal adverse effects in laboratory animals, even when administered at high concentrations [56]. However, the prolonged treatment, over approximately eight weeks, performed in this model with the higher dosage of niclosamide may have potentially produced side effects that could, in turn, diminish the efficacy of the treatment. Indeed, many side effects of niclosamide may be dose-dependent, with higher doses associated with a greater likelihood of adverse reactions as for instance elevated liver enzymes and changes in kidney function markers and hematological parameters [57]. Further experiments will be essential to explore and better understand this possibility. Indeed, in the rapidly progressive disease model represented by FUS mice, the administration of a 50 ​mg/kg niclosamide dose for approximately two weeks, demonstrates a spectrum of positive effects. While differences in the specific mouse model could contribute to variations in drug response, the pharmacokinetics of the drug, including factors such as absorption, distribution, metabolism, and elimination, can impact its efficacy. In a 2-week treatment period, as for FUS mice, the drug may not reach steady-state concentrations, and the higher dose of 50 ​mg/kg could lead to greater drug accumulation in the target tissues, resulting in enhanced therapeutic effects. In the case of SOD1-G93A mice the longer duration of treatment with a dose of 50 ​mg/kg could lead to adaptations or changes in the response to the drug. This explanation could be consistent with the results that in the first phases of the treatment niclosamide 50 ​mg/kg exert almost similar effects to 20 ​mg/kg, while in the final phase of the treatment it fails to continue to exert beneficial effects compared to untreated mice. Of importance, niclosamide effectively reduces the cytoplasmic accumulation of FUS protein in motoneurons, a key pathological hallmark in ALS [58]. Studies conducted in mouse models have shown that the cytoplasmic accumulation of FUS contributes to motoneuron degeneration [59]. Therefore, the niclosamide-mediated reduction of cytoplasmic mislocalization of FUS in motoneurons possibly contributed to their neuroprotection, as demonstrated with other treatments [28]. However, niclosamide was not able to delay denervation atrophy of hindlimb muscles during the later disease stage. This finding partly explains why the increase in surviving motoneurons corresponds to moderate improvement in motor functions in FUS mice. It is worth noting that while the human wild-type FUS model has some limitations due to its rapid disease progression [60], it serves as a valuable model for investigating the disease-modifying effects of drugs in the context of this aggressive form of the disease [28]. Additionally, it provides a suitable platform for exploring the mechanisms targeted by a drug in an *in vivo* context. Thus, the beneficial effect of niclosamide in this model suggests a potential efficacy of the drug in disease forms characterized by FUS-associated pathology.

Although the use of niclosamide in neurological diseases is still under investigation, emerging evidence suggests its potential therapeutic effects [43]. One of the limitations associated with using niclosamide in neurological disorders is the concern about its effective BBB penetrance, even though evidence related to its structure and binding to the glycoprotein P predicted its crossing. This study provided the first experimental evidence demonstrating that niclosamide traversed the BBB, particularly the blood-spinal cord barrier, following intraperitoneal injection. Notably, the concentrations in the nervous system are consistent with its IC50 for known targets, such as STAT3 [61]. Niclosamide has demonstrated a robust inhibitory effect on the expression of STAT3 and mTOR, involved respectively in inflammatory and autophagic pathways, in the spinal cord of ALS mice. Notably, dysregulation of STAT3 has been well-documented in ALS patients and animal models [62]. Moreover, compelling evidence supports the notion that inhibiting STAT3 could represent a therapeutic strategy for ALS [11].

Overall, the results obtained in this study have established that niclosamide protects motoneurons from degeneration and improves the progression of pathology, suggesting its possible clinical use for ALS. Limitations are associated with using transgenic mouse models for studying ALS. However, conducting experiments in two distinct ALS mouse models, SOD1-G93A and FUS, representative of ∼30% of fALS cases, significantly amplifies the impact and relevance of our findings. Of particular significance is the result that niclosamide therapeutic effectiveness is obtained upon administration after symptom onset. This feature enhances its potential value for future translation into clinical applications, given that most patients with sporadic forms of ALS are typically diagnosed only when they reach the symptomatic stage of the disease. Additionally, considering that a potential ALS treatment should be multifunctional and target multiple aspects of the disease, the use of niclosamide, capable of influencing different pathways, aligns with these requirements. Lastly, niclosamide well-documented safety, tolerance, and pharmacokinetic profiles would make the repurposing strategy for ALS advantageous in terms of cost, time, and effort compared to developing entirely new drugs.

## Ethics approval

Animal care procedures were conducted at the Tor Vergata University Animal Facility in accordance with the FELASA Recommendations, European Guidelines for the use of animals in research (2010/63/EU), and Italian Laws (D.L. 26/2014).

## Availability of data and materials

The datasets generated and analyzed during the current study are available in the Gene Expression Omnibus (GEO) repository under the accession number GSE249071.

## Funding

This work was founded by Fondazione AriSLA ETS (Fondazione di ricerca per la SLA ETS), ReNicALS project to SA. Research in NDA's lab is supported by #NEXTGENERATIONEU (NGEU) and founded by the Ministry of University and Research (MUR), National Recovery and Resilience Plan (NRRP), project MNESYS (PE0000006) – A Multiscale Integrated Approach to the Study of the Nervous System in Health and Disease (DN. 1553 October 11, 2022).

## Authors’ contributions

SA and NDA conceived and designed the study and wrote the manuscript. MM, IDV and CM performed the experiments. SR, PF, GN and MC analyzed the data. All authors contributed to the data analysis and interpretation. All authors read and approved the final manuscript.

During the preparation of this work, the authors used ChatGPT in order to improve language and readability. After using this tool, the authors reviewed and edited the content as needed and take full responsibility for the content of the publication.

## Declaration of competing interest

The authors declare that they have no known competing financial interests or personal relationships that could have appeared to influence the work reported in this paper.
