# Supplementary material for: Neuroprotective effects of niclosamide on disease progression via inflammatory pathways modulation in SOD1-G93A and FUS-associated amyotrophic lateral sclerosis models
Source: Neurotherapeutics. 2024 Mar 15;21(3):e00346. doi: 10.1016/j.neurot.2024.e00346 (PMC11070272; doi:10.1016/j.neurot.2024.e00346)
Supplement: Multimedia component 1 [file mmc1.docx]

**Supplementary material**

**Figure S1. Niclosamide slightly ameliorates disease progression in female SOD1-G93A mice.** Neurological scores (a), grip test (b) and rotarod performance (c) were slightly ameliorated in niclosamide 20 mg/kg (n=10, green) compared to vehicle-treated SOD1-G93A mice (n=10, blue). Data represent means ± S.E.M. Statistical significance was calculated by ANOVA, *p < 0.05, **p < 0.01, and ***p < 0.001 Veh *vs* Nic. (d) Kaplan–Meier curve of SOD1-G93A mice showing increased survival in niclosamide group as compared to vehicle group. Statistical significance was calculated by log-rank test referred to vehicle, **p<0.01.

**Figure S2. Niclosamide at higher dose ameliorates disease progression in SOD1-G93A mice but not affects survival.** Neurological scores (a), wire test (b) and rotarod performance (c) were significantly ameliorated in niclosamide 50 mg/kg (n=8, red) compared to vehicle-treated SOD1-G93A mice (n=12, blue). Data represent means ± S.E.M. Statistical significance was calculated by ANOVA, *p < 0.05, **p < 0.01, and ***p < 0.001 Veh *vs* Nic. (d) Kaplan–Meier survival curves of SOD1-G93A mice in niclosamide group as compared to vehicle group. Statistical significance was calculated by log-rank test referred to vehicle, **p<0.01.

**Figure S3. Niclosamide inhibits mTOR and STAT3 signalling pathways.** Representative western blots (a) and quantification (b) of p-mTOR and STAT3 in Ntg, vehicle- and 20 mg/kg niclosamide-treated SOD1-G93A mice (n=4 mice/group). GAPDH was used as a loading control. Data represent means ± SEM. Statistical significance was calculated by ANOVA, **p*< 0.05.

**Figure S4. Heatmap of differentially expressed genes in the spinal cord of niclosamide treated SOD1-G93A mice at symptomatic phase of the disease.** Hierarchical clustering heatmap of differentially expressed genes of niclosamide (nic) compared to vehicle (veh) SOD1-G93A mice with a p-value < 0.01 and fold change greater than 1.2 at the symptomatic phase of the disease. n=3 animals/group. The heatmap shows the z-scores for each gene across samples: green and red colours represent gene downregulation and upregulation, respectively.

**Figure S5. Niclosamide decreases microgliosis in lumbar spinal cord of FUS mice.** (a) Representative confocal images of lumbar spinal cord sections of vehicle- and 50 mg/kg niclosamide-treated FUS mice immunolabeled with Iba1 (red). Scale bar: 100 μm. Quantification of Iba1 staining in hemisections of both groups. (b) Representative confocal images of lumbar spinal cord sections of vehicle- and 50 mg/kg niclosamide-treated FUS mice immunolabeled with GFAP (green). Scale bar: 100 μm. Quantification of GFAP staining in hemisections of both groups. Data are expressed as means ± SEM (at least 4 sections for each animal). Statistical significance was calculated by student’s t-test referred to vehicle-FUS group, ***p<0.001.


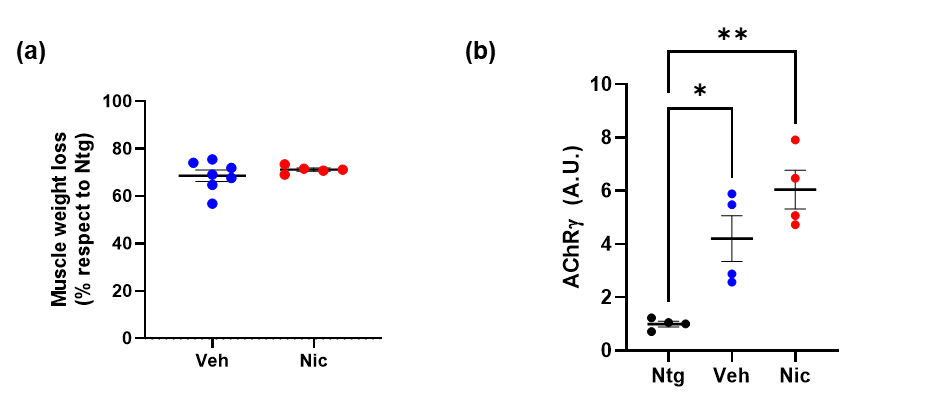


**Figure S6. Niclosamide** **has no effect on muscle denervation atrophy.** (a) Muscle weight of vehicle and niclosamide-treated FUS mice. (b) Real-time qPCR for AChRy mRNA transcripts in GCM muscle of vehicle and niclosamide-treated FUS mice compared to Ntg littermates. Data represent mean ± SEM. Statistical significance was calculated by one-way ANOVA with Tukey’s post-analysis, * p< 0.05, **p<0.01 (n=3/4 animals for group).

**Supplementary Table 1.** List of differentially expressed genes (DEGs) in the spinal cord of niclosamide treated SOD1-G93A mice compared to vehicle-treated mice with a p-value < 0.01 and fold change greater than 1.2, both at the end stage and at the symptomatic phase of the disease.
